# Specialized expertise among healthcare professionals in palliative care - A scoping review

**DOI:** 10.1186/s12904-024-01498-0

**Published:** 2024-07-13

**Authors:** Fleur Godrie, Ingrid van Zuilekom, Bregje Onwuteaka-Philipsen, Harmieke van Os-Medendorp, Linda Schoonmade, Suzanne Metselaar

**Affiliations:** 1 AmsterdamUMC Department of Ethics, Law and Humanities, Expertise Center for Palliative Care, Amsterdam, Hoofddorp The Netherlands; 2 AmsterdamUMC, Expertise Center for Palliative Care Research group Smart Health, School of Health Saxion, University of Applied Sciences, Amsterdam, Hoofddorp The Netherlands; 3 AmsterdamUMC, Department Public and Occupational Health, Expertise Center for Palliative Care, Amsterdam, Hoofddorp The Netherlands; 4 Inholland University of Applied Sciences, Faculty of Health, Sports and Social Work, Amsterdam, The Netherlands & Spaarne Gasthuis Academy, Amsterdam, Hoofddorp The Netherlands; 5 University Library, Vrije Universiteit Amsterdam, Amsterdam, Hoofddorp The Netherlands

**Keywords:** Palliative care, Specialized expertise in palliative care, Generalist-plus-specialist model, Professional competencies

## Abstract

**Background:**

The generalist-plus-specialist palliative care model is endorsed worldwide. In the Netherlands, the competencies and profile of the generalist provider of palliative care has been described on all professional levels in nursing and medicine. However, there is no clear description of what specialized expertise in palliative care entails, whereas this is important in order for generalists to know who they can consult in complex palliative care situations and for timely referral of patients to palliative care specialists.

**Objective:**

To gain insight in the roles and competencies attributed to palliative care specialists as opposed to generalists.

**Methods:**

A scoping review was completed based on PRISMA-ScR guidelines to explore the international literature on the role and competence description of specialist and expert care professionals in palliative care. Databases Embase.com, Medline (Ovid), CINAHL (Ebsco) and Web of Science Core Collection were consulted. The thirty-nine included articles were independently screened, reviewed and charted. Thematic codes were attached based on two main outcomes *roles* and *competencies*.

**Results:**

Five roles were identified for the palliative care specialist: care provider, care consultant, educator, researcher and advocate. Leadership qualities are found to be pivotal for every role. The roles were further specified with competencies that emerged from the analysis. The title, roles and competencies attributed to the palliative care specialist can mostly be applied to both medical and nursing professionals.

**Discussion:**

The roles and competencies derived from this scoping review correspond well with the seven fields of competence for medical/nursing professionals in health care of the CanMEDS guide. A specialist is not only distinguished from a generalist on patient-related care activities but also on an encompassing level. Clarity on what it entails to be a specialist is important for improving education and training for specialists.

**Conclusion:**

This scoping review adds to our understanding of what roles and competencies define the palliative care specialist. This is important to strengthen the position of the specialist and their added value to generalists in a generalist-plus-specialist model.

**Supplementary Information:**

The online version contains supplementary material available at 10.1186/s12904-024-01498-0.

## Background

Interprofessional collaboration is considered to be key to the provision of high-quality palliative care [1]. In addition to generalist healthcare professionals, palliative care specialist professionals play a crucial role in providing high-quality care to individuals with life-limiting illnesses and vulnerability.

The generalist-plus-specialist palliative care model is the main model endorsed worldwide [2]. Ever since the value of palliative care started to become recognized in the 1990s, specialists are called for to take care of palliative care needs. The demand for palliative care specialists increased rapidly as timely palliative care consultations have proven to improve the quality of care and are beneficial for healthcare costs [2–4]. However, for the (increasing) amount of patients with palliative care needs, it is impossible to provide specialized palliative care to all of them [5, 6]. Therefore, the generalist-plus-specialist palliative care model should distinguish general palliative care from specialist palliative care. In order for the generalist-plus-specialist palliative care model to be a sustainable model, the palliative care specialist skill set to manage more complex palliative care cases should be defined [2, 7, 8].

In the Netherlands, palliative care is provided by means of a mixed model of generalist and specialist care. In the Dutch quality framework on palliative care (NQFPC) the role of the specialist and also the expert is recognized next to the role of generalist palliative care [9, 10]. In short, the model entails that palliative care is primarily provided by generalist healthcare professionals (physicians, vocational and bachelor nurses, clinical nurse specialists and other allied health professionals) who are not considered specialists in palliative care [2, 9]. In fact, all healthcare professionals are expected to be generalists in palliative care, i.e. they should be able to provide palliative care according to national quality standards and guidelines [10]. Referral to palliative care specialists and experts will take place in case of complex situations, such as patients with multiple chronic diseases [8]. Involvement of palliative care specialists is deemed to be important in delivering appropriate and high-quality care in complex cases. The competencies and profile of the generalist provider of palliative care has been described [9]. However, as of yet, there is no clear definition of what is understood by specialists and experts in palliative care, and how this expertise relates to the role of the generalist. This pertains to all nursing and medical professionals.

A definition of specialized expertise in palliative care is important in order for generalists to know who they can consult in complex palliative care situations and for patients to be referred to palliative care specialist in time. In this article, the international literature is reviewed to assess how specialized expertise in palliative care is understood worldwide. This scoping review is part of a larger study that seeks to establish consensus on the role definition and assignment of specialist and expert palliative care providers in the Dutch palliative care system.

The objective of this scoping review is to define and describe the roles and competencies of specialists as opposed to generalists in palliative care, and that of experts in palliative care.

## Method

### Search strategy

A scoping review was completed based on PRISMA-ScR guidelines [11, 12]. To identify potentially relevant documents, the following bibliographic databases were searched in July 2022: Embase.com, Medline (Ovid), CINAHL (Ebsco) and Web of Science Core Collection. The search strategies were drafted by professional clinical librarian L.S and further refined through team discussion. The search strategy consisted of two concepts. First, terms for palliative care such as “generalist palliative care” “specialist” and “expertise” were searched. Second, terms for role description and requirements on meta-level were searched such as “characteristics”, “competencies” and “medical education”. These concepts were combined in the search strategy. The complete search strategy is included in Additional file 1 Appendix 1.

Search results were limited to those published after the year 2010, to ensure that included studies were representative of modern palliative care. Only published studies were reviewed thus ethics approval was not required. Grey literature was not included. Only English (translated) search results with human subjects were included in abstract review. Preferred reporting items for systematic reviews and meta-analyses (PRISMA) guidelines were followed to ensure methodological best practices [11, 13].

### Study selection

The final search resulted in 13881 records. These were exported into EndNote, and duplicates were removed by L.S. Titles and abstracts were reviewed for eligibility. F.G and I.v.Z screened the titles and abstracts, based on the following inclusion criteria: manuscripts (1) assess palliative care; and (2) report on palliative health care system or program; or (3) report on either (or both) of the outcomes role and competencies. Studies on graduate education and RCTs were excluded as we did not seek empirical evidence for, for instance, the added value of palliative care specialists to the quality of care here. Rather, we focus on how specialized expertise is generally understood in the literature. Therefore, we also excluded single case studies. After the abstracts were included, F.G and I.v.Z proceeded to full text review. At full text review, S.M was consulted to review and resolve any inclusion disagreement.

### Analysis

Thematic component analysis was used to describe the palliative care specialist components in international palliative care systems [14]. A data-charting table was jointly developed by the F.G and S.M to determine which variables to extract from the articles. F.G, I.v.Z and S.M independently charted the data. The results were discussed among all authors, the data charting table was continuously updated in an iterative process.

For the deductive thematic synthesis, F.G attached thematic codes to the most important segments that were collected from the data [15]. The segments were coded based on the two main outcomes that were derived from the articles, namely: *roles* and (related) *competencies* of the palliative care specialist. F.G and S.M discussed the coded segments. Consensus was reached among all authors on the included segments. The roles that emerged out of the literature formed the structure of the results section. All authors agreed on the fact that the theme ‘leadership’ was a main outcome of the scoping review. The authors have considered presenting ‘leader’ as one of the roles in the result section. However, it was finally decided, as leadership qualities are relevant in the other roles as well, to describe leadership as a quality that runs throughout all five roles of the palliative care specialist that were identified.

The included studies were classified by the two main outcomes: roles and competencies for palliative care specialists in nursing and medical professions. Data extracted, independently and verified by a second and third reviewer, included the study type, study population and the themes: leadership, caretaking, consultancy, educating, researching, advocating.

## Results

### Study characteristics

**Fig. 1:**
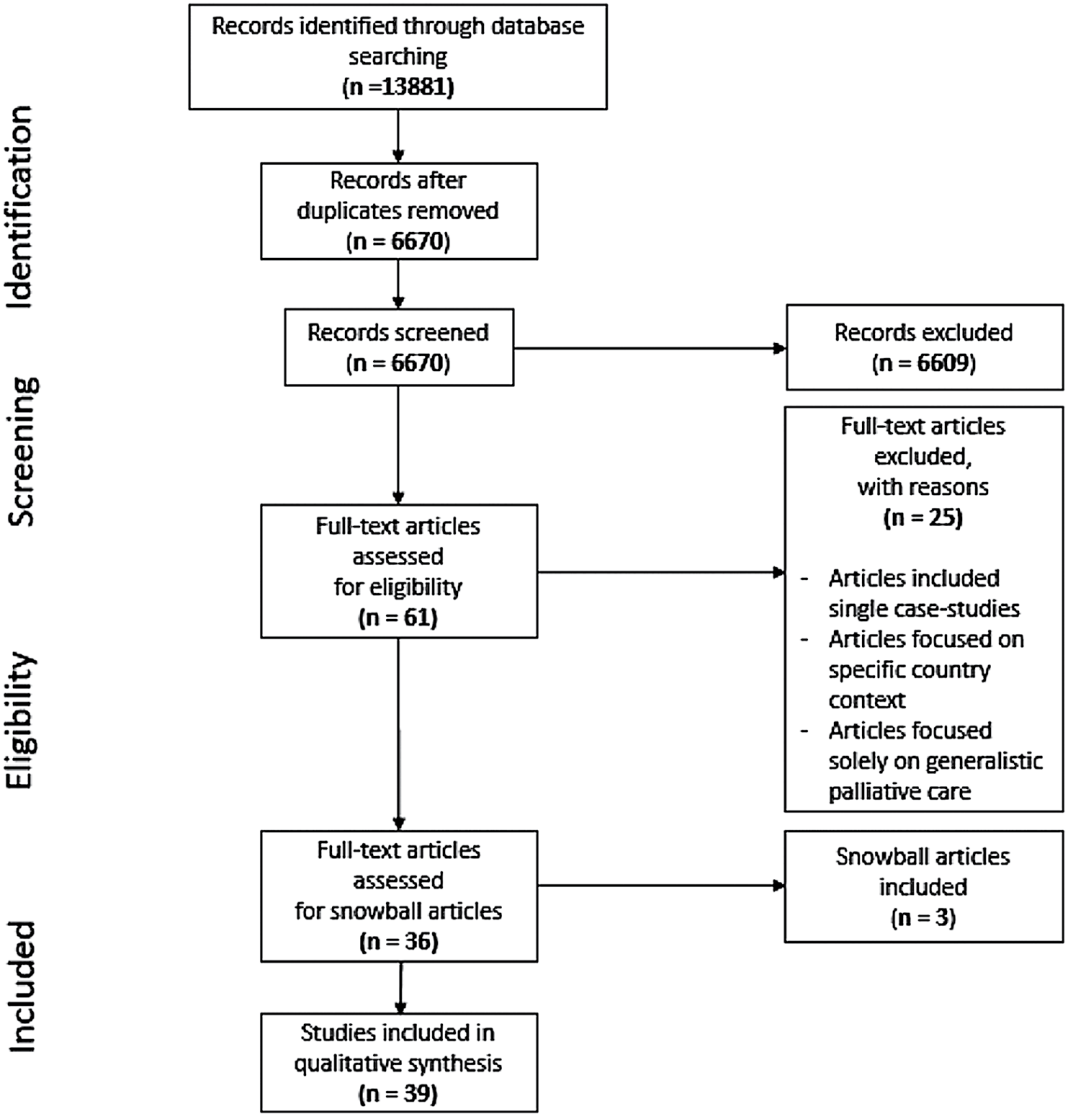
PRISMA flow diagram

Thirty-nine articles were included in this scoping review (Fig. 1). Eleven out of the thirty-nine articles solely focus on physicians as palliative care specialists, fourteen articles focus on nurses as specialists and thirteen articles focus on healthcare professionals with specialized expertise in palliative care in general. Research designs include Delphi-studies, literature reviews and qualitative research designs. The articles cover palliative care in different countries: seven are set in the USA [2, 16–21], five in the United Kingdom [22–26], five in Australia [27–31], four in Ireland [7, 8, 32, 33], three in Finland [34–36], two in the Netherlands [10, 37], two in India [38, 39] and two in Italy [40, 41]. The remaining selected articles are based in Canada [42], China [43], Germany [44], Japan [45], Korea [46], New Zealand [47], Sweden [1], Switzerland [48] and Taiwan [49]. Included study characteristics are presented in Additional file 2: Table S1.

### Expert

Only four out of the thirty-nine articles included in this scoping review mention ‘expert’ as a term for a professional with specialized expertise in palliative care. In neither of these articles, however, the expert was a *formal* designation, like ‘generalist’ or ‘specialist’. Rather, it was mentioned referring to the ‘medical expertise’ of a medical specialist, or in the context of an ‘expert panel’ as part of a research strategy. However, it was not recognized as a formal title next to - or beyond the title of the palliative care specialist [16, 17, 41, 46]. Accordingly, it was not further defined or related to certain roles and competencies.

### Specialist

The selected articles discuss a variety of roles and competencies that define the palliative care specialist. Leadership qualities run throughout these roles. We found little distinction between the nursing and medical professions. In general, we found that palliative care specialties constitute expertise additional to and beyond generalist palliative care. A palliative care specialist is not always needed; specialists are referred to if care needs exceed the capabilities of generalists. For instance, in case of complex (bereavement) symptoms, intolerable pain symptoms and multimorbidity. Furthermore, palliative care is considered an *integrated care specialty*. It is therefore considered to be ideal when both nursing and medical professionals are part of interdisciplinary palliative care teams.

### Leadership qualities of palliative care specialists

We found leadership qualities to be pivotal for palliative care specialists. We did not identify leadership as a separate role. Rather, it runs throughout all roles for the palliative care specialist. Leadership is generally described through three competencies. The first one is the ability to establish an holistic overview of the care and cure situation and perform holistic assessments during the patient-journey [1, 16, 38, 40, 41]. Secondly, the specialist is able to navigate the care process, facilitating the complex transitions that need to take place during the patient-journey; also referred to as ‘case-manager’ among palliative care specialist nurses [16, 24, 38, 40, 41, 48]. The last one is the ability to lead interdisciplinary care teams and to ensure visibility of these teams, as timely referral to these teams is considered to support the continuity and quality of care [1, 21].

The expertise that the palliative care specialist can provide in addition to generalist palliative care is best described in terms of the different roles the specialist can perform, and the related competencies the specialist should have. From our analysis of the selected articles, five roles emerge:

- Care provider
- Care consultant
- Educator
- Researcher
- Advocate

The roles are further described in this section. Under each role, several competencies are subsumed. We found that the roles and competencies attributed to the specialist can mostly be applied to both medical and nursing professionals. If the role or competence is specifically assigned to either medical or nursing professionals, it is described as such in this section.

Table 1 provides an overview of the five roles and related competencies. Leadership was not identified as a separate role as it runs throughout all roles for palliative care specialists. Many of these competencies apply to more than one role. For example, being able to carry out advance care planning (ACP) is attributed to the role of both care consultant, and care provider. The findings of this scoping review also show that ‘exceptional communication skills’ are recognized as a competence that applies to different roles the palliative care specialist performs.

**Table 1:** Overview of roles and competencies for palliative care specialists*

Role	Competence
Care provider	- performing holistic assessments of the patient-journey [1, 16, 38, 40, 41] - managing complex symptoms [2, 16, 25, 26, 28, 32, 34–36, 41, 42, 45, 46] - supporting complex bereavement [8, 19, 28, 33, 45, 46] - carrying out advance care planning (ACP) [25, 35, 39, 48] - having exceptional communication skills [19, 28, 38, 43]
Care consultant	- facilitating and managing complex transitions during the care process [16, 24, 38, 40, 41, 48] - providing specialized advice to patient and family [25, 28, 33, 42, 45] - providing specialized advice to generalists (care team) [8, 10, 21, 28, 42] - supporting complex bereavement [8, 19, 28, 33, 45, 46] - having exceptional communication skills [19, 28, 38, 43] - carrying out advance care planning (ACP) [25, 35, 39, 48] - providing accurate advice and care on ethical issues [28, 38, 45]
Educator	- disseminating information about palliative care to colleagues [1, 10, 18, 28, 37, 40] - bed-side teaching [20, 28–30, 5, 37, 46] - coaching generalists in palliative care [18, 20, 28–31, 35, 37, 46]
Researcher	- contributing to palliative care research [8, 25, 32, 35, 45, 46]
Advocate	- contributing to awareness raising for palliative care [34, 37] - advocating for the needs and goals of the patient and family in (complex) palliative care processes [8]

*Leadership was not identified as a separate role as it runs throughout all roles for palliative care specialists

### Care provider

The palliative care specialist performs the role of care provider when the patient’s care is too complex or challenging for a generalist care provider [2, 16, 25, 26, 28, 32, 34–36, 41, 42, 45, 46]. Generalists typically refer patients to palliative care specialists when decisions on multi-morbidity treatments within the patient journey (over different settings of care) become too complex, or in cases of severe pain management. The nurse palliative care specialist is often called a ‘case manager,’ who manages the care process for the patient over different settings of care, holistically oversees patient-journeys and manages complex palliative care needs of the patient between care disciplines [20, 25, 30, 35, 48].

The medical palliative care specialist needs to be able to have specified (pharmacological) knowledge and expertise in physical care, psychological care, social care as well as spiritual care and perform distinct clinical practice [17, 25, 26, 28, 31, 34, 35, 45, 46, 49]. The specialist needs to discuss and make decisions on burdensome treatments and complex symptom management. As was stated in the article of Ballou, the specialist should be able to manage and coordinate complex care cases: *“Specialty palliative care includes - learning to manage refractory symptoms and - coordinate complex care demands.”* [17](P803). By assessing and managing complex symptoms, such as severe pain management or complex bereavement care, the overall goal of the specialist should be to be able to improve the quality of life of the patient and family [2, 16, 31, 41]. The management of complex palliative care requires specific competencies, such as:

*“… real time decision making, coordinating (onward) referral to other specialists, extensive communication skills, introducing difficult conversations and balancing hope with realism.”* [25](p.251).

Advance care planning conversations with the patient and family cover sensitive topics such as the patient’s prognosis and the process of dying [38]. In all of the roles the specialist performs, ACP forms a base for continuous holistic assessment of the patient-journey and communication with the patient and family [25, 35, 39, 48]. In order to perform these holistic assessments, the specialist needs to have exceptional communication skills [19, 28, 38, 43]. The specialist needs to specifically be able to recognize and respond to reactions of grief and loss of the patient and family. In case of complex bereavement symptoms in patients and families, bereavement support is an important part of the specialist’s services as care provider [8, 19, 28, 33, 45, 46]. In the article of Forbat [28], communication and listening competencies are therefore described as typical skills for palliative care specialists:

*“To have specific communication skills, particularly around the ability to discuss and plan for death and dying, with compassion and empathy, having exceptional listening skills and having the ability to manage conflict.”* [28](p.7).

### Care consultant

A palliative care specialist performs the role of *care consultant* by giving advice and guidance. The specialist gives advice to and leads (an interdisciplinary team of) caregivers in specific patient cases where generalist care is not sufficient. The specialist should for instance be able to guide generalists through consultation and transfer of care:

*“Palliative care specialists should support generalists through consultation palliative care and transfer of care (i.e. care transferred prior to death to a specialist palliative care physician) where indicated.*” [42](p.1334).

The consulting role can also be directed at the patient and family when generalists refer to specialists for specialized advice on the palliative care process [25, 28, 33, 42, 45]. The specialist needs to cooperate with – and be visible for generalists in order to be referred to in time [8, 10, 21, 28].

In complex care cases, physical and emotional symptoms are often based on multi-morbidity and emotional family dynamics, and have to be taken care of simultaneously [1, 28]. In order to guarantee person-centered care, in addition to the generalist care protocol, the specialist recognizes challenges faced by the patient and family and advices them in their adaptations to the changes in the palliative care process [33, 35].

The palliative care specialist in the role of care consultant has especially an important role in ACP [25, 35, 39, 48]. The specialist provides effective and efficient person-centered planning and service to patients in the role of care consultant [27, 34]. Furthermore, they combine the shared impressions within the interdisciplinary care team and discusses the patient and family [1]. The specialist should be able to initiate and review the patients’ advance care plan, based upon the patient and family’s expressed values, goals, needs and preferences [38].

Next to supporting the family as a unit of care, the specialist is able to reflect on oneself as a care giver working in a team and leading an interdisciplinary care team, as well as on the individual care givers that form the interdisciplinary care team [38, 45]. The specialist has a coaching role towards colleagues, negotiating and empowering generalist nurses in the palliative care process [37, 48]. In this continuous cycle of reflection during the care process, the specialist should support the team by providing psychological care for oneself and the care givers in the care team [45].

In the role of consultant, the specialist needs to be able to understand ethical themes and values related to palliative care, such as autonomy and dignity, and have advanced knowledge of identified dying in order to provide moral advice [28, 45]. The specialist needs to be able to provide accurate advice and care on ethical issues that may occur and is expected to have knowledge of the ethical and legal framework that applies to complex palliative care cases [38]. The specialist has the communication skills to work in – and lead an interdisciplinary team of caregivers in ethically themed care cases, and communicates directly to the patient and family when specialized advice on their moral questions in the care process is needed [28, 38, 45].

### Educator

In general, a palliative care specialist is considered to be an interprofessional educator and bedside teacher to generalist care professionals around them [1, 10, 18, 28, 37, 40]. The main task of a specialist as an educator is to convey knowledge and skills about palliative care to colleagues, to mentor generalists in palliative care, and to keep generalists up to date on developments in palliative care practice [20, 28–30, 35, 37, 46]. For instance, ACP is an acknowledged part of palliative care practice for healthcare professionals in general, yet in complex care cases, educating colleagues about ACP is specifically a task for the specialist.

Another task is to keep generalists aware of complex care cases where specialist care is needed, also in order for timely referral to specialists to take place [8, 10].

Articles focused on specialist clinicians describe that interprofessional education forms an integral element of their daily job [18, 31, 46]. Palliative care teams are typically interdisciplinary and therefore require structural information sharing between physicians and nurses and other care professionals involved [40]. As a specialist clinician, you ‘share the load’ with your colleague care professionals and provide them with education for their future skill development [31].

*“Specialists should focus on patients who likely benefit from their specific palliative care skills and in the meantime use their time to train basic palliative care skills to clinicians who are caring for these patients.”* [18](p.565).

As was stated in the article of Carroll [18], the role of educator requires the ability to combine advanced palliative care competencies with training skills on basic palliative care practice.

### Researcher

Engaging in palliative care research is considered to be an important aspect of being a palliative care specialist [8, 25, 32, 35, 46]. Yet, perspectives on how specialists should engage in research diverge. This could pertain to initiating or conducting research, but also to participating in research, or making sure you are up-to-date with scientific developments in the field of palliative care [8, 33, 40, 46]. For instance, in the article of Sakashita this ‘research responsibility’ is described as: *“the ability to contribute to the development of palliative care by being involved in education and research as well as constantly updating knowledge as a palliative care specialist.”* [45](p.33).

### Advocate

The role of advocate was only found for the palliative care specialist nurses, not for physicians [8]. The role of advocate can be performed on two levels. On one level, through raising awareness on (high quality) palliative care in a broader (societal) context. For example, the nurse palliative care specialist who engages in networking occasions to advocate for palliative care [34, 37]. On another level, the role of advocate can be performed by representing (or being the spokesperson of) the patient and family in (complex) palliative care processes:

*“The role was described as educative and supportive: ‘about advocacy’ for the patient and family, a sense of continuity of care, and was a resource to support the care for patients with complex need.”* [8](P4).

Through this manner of advocating for the palliative care of specific patients, the specialist can stand for the needs and goals of individual patients and their families and subsequently use these unique patient-journeys as examples for (high quality) palliative care in general.

## Discussion

This review of the international literature aimed to map and describe the role and competencies attributed to specialists in palliative care. For both the nursing and medical professionals palliative care, five roles were identified, namely; ‘care provider’, ‘care consultant’, ‘educator’, ‘researcher’ and ‘advocate’. Different competencies were related to these roles, whereas leadership qualities were considered to be pivotal in roles identified for the palliative care specialist. Based on the fact that there was no information found on the identified role of expert palliative care, we may conclude that palliative care system models outside of the Netherlands do not recognize the role of ‘expert’ in palliative care, at least not on a formal level. The identification of three levels of palliative care provision might be unique for the Dutch care context. Yet, as there is no clarity and consensus yet on what specialized expertise in palliative care precisely entails in the Netherlands – which was an important reason to conduct this scoping review – it remains to be seen how and whether these three levels will be maintained.

This review does not include a specific search for articles that point out the distinction between generalists and specialists in palliative care. Rather, the search strategy led to articles that highlight what is expected of specialists in palliative care. The NQFPC prescribes that all health care professionals are expected to provide general palliative care according to national standards and guidelines [9]. The NQFPC describes all basic palliative care skills for education for nursing and medical professionals. However, the roles of the palliative care specialist that we identified here go above and beyond what is expected of generalists. The specialist is also expected to perform these roles in highly complex care situations. Some roles, such as that of educator, specifically address how specialists should relate to generalists. The collaboration between generalists and specialists in palliative care practice could be further explored by comparing the roles and tasks that are expected from them. Further research could focus more precisely on how the roles of generalists and specialists relate in providing good palliative care in practice.

### Distinctions between the nurses and medical professions

Interestingly, almost every role and competence that is expected of and required from the palliative care specialist is applicable to medical as well as nursing professionals. Only a few differences came up between nursing and medical professions at palliative care specialist level. For instance, the role of advocate solely emerged in articles that focused on nursing professions [8, 34, 37]. Yet, the description of the role included competencies, such as ‘awareness raising’, that may just as well be performed by medical professionals. Notably, in the article of Kang, the competencies of a palliative care specialist were differentiated for the nursing and the medical professional [46]. The competence specifically assigned to medical professionals included ‘PC service management’, ‘ACP’, and ‘physical care and treatment’. As for the nurses, the assigned competencies include ‘pain and symptom management’ and ‘quality assurance’ [46].

Whereas the identified roles and competencies are almost similar for the nursing and medical professions, the interpretation of the role does depend on the responsibilities and mandates appropriate to the laws and regulations of the profession in a specific country. Yet, this similarity suggests that the palliative care specialist is an overarching title and integrated position within the health care system.

### Comparison with the CanMEDS roles

In order to relate the roles and competencies in this scoping review to existing education curricula, we studied the CanMEDS roles. The CanMEDS (Canadian Medical Education Directions for Specialists) describe seven fields of competence for medical and nursing professionals in health care [50]. These roles were found to partly overlap with the roles and competencies we have defined for palliative care specialist.

For example, the CanMEDS recognize the role of ‘Medical expert’ (and decision maker) for physicians, and ‘Care provider’ for nurses and further define these roles with competencies such as: perform patient centered clinical assessment, working in a team of care professionals, and provide timely consultation to the patient and family. These are three competencies that are derived from the included articles in this scoping review as well. In the case of our review, they are attributed to roles very similar to the CanMeds roles, i.e. the role of care provider and care consultant.

As is stated and visualized in Table 1, the competencies attributed to the roles are not fixed to those roles; they run through the different roles of the palliative care specialist. The CanMEDS further describe the role of ‘communicator’, whereas in this scoping review, we recognized exceptional communication skills as a competence that applies to many different roles the palliative care specialist performs. The following four of the CanMEDS role definitions are mostly comparable to the roles we recognize in this scoping review: the role of ‘Leader’ shows similarities with the leadership qualities recognized as a main finding in this scoping review, covering the overviewing and guiding aspect of being a palliative care specialist. The role of ‘Health advocate’ shows similarities with the role definition of advocate in this scoping review, however it only covers the aspect of advocating for the needs and goals of the patient and family. The competence of networking and awareness raising for palliative care that is part of performing the role of advocate in this scoping review, is applied to the role of ‘Professional’ in the CanMEDS guide. Finally, the CanMEDS guide describes the role of ‘Scholar’, mostly focusing on the continuous enhancement of professional activities through ongoing learning. Here we see a combination of two roles recognized in this scoping review, namely the role of Educator and Researcher.

Remarkably, the role of researcher as palliative care specialist was mentioned in seven out of thirty-nine included articles [8, 25, 32, 35, 40, 45, 46]. However, none of these articles provided a specific description of this role. It is therefore still not clear what the role of researcher exactly entails. Does this mean that specialists should initiate or conduct research? Or should they be involved in scientific research? Or is making a contribution to palliative care guidelines, for instance by participating in a working group, also sufficient? Thus, it is not clear how specialists should perform this role of researcher. The role description of ‘Scholar’ in the CanMEDS could help further define the role of researcher for palliative care specialist.

Hence, from our comparison of the findings of our review to the CanMEDS roles, it has become clear that they largely overlap or correspond, and might further enrich and refine each other in the field of palliative care.

### Implications for the generalist-plus-specialist palliative care model

The generalist-plus-specialist palliative care model expects all healthcare professionals to have a generalist understanding of palliative care, enabling them to initiate and provide palliative care [2, 9]. In most palliative care models, this generalist expertise is not expected from all healthcare professionals, at least not explicitly. In the Netherlands, however, this is the case. The competencies needed for all healthcare professionals to provide generalist palliative care are well-described [9]. However, unlike in many other countries, the palliative care specialist is not recognized as a medical specialism in the Netherlands. In addition, the conditions and competencies required for specialists are not yet well-defined or agreed upon. It is therefore recommended to assess to which extent the roles and competencies of palliative care specialist as identified in this review are applicable to specialists palliative care in the Netherlands. This would support the quality of palliative care and the strength of the generalist-plus-specialist model in the Dutch palliative care context. First and foremost, it would help to strengthen curricula for education and training for specialists in palliative care.

### Strengths and limitations

A strength of this review is that the selection of articles was not restricted to specific palliative care contexts similar to the Dutch one. Furthermore, we included articles on both nursing and medical professions in all care settings in which palliative care is given. This led to a rich and saturated yield of descriptions of specialized expertise in palliative care.

A limitation is the restriction to scientific, peer-reviewed literature in this scoping review. We decided not to include ‘grey literature’ such as education curricula documents, although these documents often specify education requirements and competencies. Future research could focus on reviewing this kind of material, as it could be of added value to the present findings.

## Conclusions

This scoping review provides an overview of roles and competencies that define and describe the palliative care specialist. For both nursing and medical professionals in palliative care, five roles were identified; ‘care provider’, ‘care consultant’, ‘educator’, ‘researcher’ and ‘advocate’. Leadership qualities were found to be pivotal in all roles identified for the palliative care specialist. This overview creates a broader and more lucid understanding of the added value of the palliative care specialist, and helps to position specialists in different healthcare settings. Finally, clarity about the required roles and competencies of palliative care specialists is pivotal to strengthen the curricula of palliative care training and education.

### Electronic supplementary material

Below is the link to the electronic supplementary material.

Supplementary Material 1

Supplementary Material 2

## Data Availability

No datasets were generated or analysed during the current study.

## Abbreviations

ACP: Advance Care Planning
CanMEDS: Canadian Medical Education Directions for Specialists
PRISMA-ScR: Prevention Recovery Information System for Monitoring and Analysis – Scoping Review
NA: Not applicable
