# Supplementary material for: Specialized expertise among healthcare professionals in palliative care - A scoping review
Source: BMC Palliat Care. 2024 Jul 13;23:170. doi: 10.1186/s12904-024-01498-0 (PMC11245811; doi:10.1186/s12904-024-01498-0)
Supplement: Supplementary file 1 — Supplementary Material 1 [file 12904_2024_1498_MOESM1_ESM.docx]

### Additional file 1: Appendix 1: The complete search strategy

### Results 20 July 2022

| Database | Results 20 July 2022 |
| --- | --- |
| Embase.com | 3604 |
| Medline (Ovid) | 3263 |
| Cinahl (Ebsco) | 3681 |
| Web of Science Core Collection | 3333 |
| Total | 13881 |
| After deduplication | 6670 |

### Embase.com History and Search Details - 20 July 2022

| **Search** | **Query** | **Results** |
| --- | --- | --- |
| #5 | #4 NOT (undergraduate OR medical-student* OR nurs*-student* OR family-care* OR informal-care* OR covid OR oncol*):ti | 3,604 |
| #4 | #3 NOT (('adolescent'/exp OR 'child'/exp OR adolescent*:ti,ab,kw,kw OR child*:ti,ab,kw OR schoolchild*:ti,ab,kw OR infant*:ti,ab,kw OR girl*:ti,ab,kw OR boy*:ti,ab,kw OR teen:ti,ab,kw OR teens:ti,ab,kw OR teenager*:ti,ab,kw OR youth*:ti,ab,kw OR pediatr*:ti,ab,kw OR paediatr*:ti,ab,kw OR puber*:ti,ab,kw ) NOT ('adult'/exp OR 'aged'/exp OR 'middle aged'/exp OR adult*:ti,ab,kw OR man:ti,ab,kw OR men:ti,ab,kw OR woman:ti,ab,kw OR women:ti,ab,kw)) NOT ('conference abstract'/it OR 'conference review'/it) AND [2010-2022]/py | 4,157 |
| #3 | #1 AND #2 | 10,491 |
| #2 | ('specialization'/exp OR 'nurse practitioner'/exp OR 'caregiver'/exp OR 'physician'/exp) AND ('nursing role'/exp OR 'competence'/exp OR 'education'/de OR 'continuing education'/exp OR 'medical education'/exp OR 'nursing education'/exp) OR ((specialist* OR expert* OR non-expert* OR nurs* OR caregiver* OR care-giver* OR physician*) NEAR/3 (role OR roles OR defin* OR disting* OR characteri* OR skill* OR knowledge OR competen* OR scope-of-practice OR education* OR training*)):ab,ti,kw | 275,841 |
| #1 | 'palliative therapy'/exp OR 'palliative nursing'/exp OR 'terminal care'/exp OR (palliative OR terminal OR hospice OR limited-life OR end-of-life):ab,ti,kw | 730,812 |

### Medline (Ovid) History and Search Details – 20 July 2022

| **Search** | **Query** | **Results** |
| --- | --- | --- |
| #6 | 5 not (undergraduate OR medical-student* OR nurs*-student* OR family-care* OR informal-care* OR covid OR oncol*).ti | 3,263 |
| #5 | 4 and 2010:2023.(sa_year). | 3,817 |
| #4 | 3 NOT ((Adolescent/ OR Child/ OR Infant/ OR adolescen*.ti,ab,kf. OR child*.ti,ab,kf. OR schoolchild*.ti,ab,kf. OR infant*.ti,ab,kf. OR girl*.ti,ab,kf. OR boy*.ti,ab,kf. OR teen.ti,ab,kf. OR teens.ti,ab,kf. OR teenager*.ti,ab,kf. OR youth*.ti,ab,kf. OR pediatr*.ti,ab,kf. OR paediatr*.ti,ab,kf. OR puber*.ti,ab,kf.) NOT (Adult/ OR adult*.ti,ab,kf. OR man.ti,ab,kf. OR men.ti,ab,kf. OR woman.ti,ab,kf. OR women.ti,ab,kf.)) | 6,051 |
| #3 | 1 and 2 | 6,345 |
| #2 | ((exp Specialization/ or exp Nurse Practitioners/ or exp Caregivers/ or exp Physicians/) and (exp role/ or exp Professional Competence/ or exp "Education, Professional"/)) OR ((specialist* OR expert* OR non-expert* OR nurs* OR caregiver* OR care-giver* OR physician*) ADJ4 (role OR roles OR defin* OR distin* OR characteri* OR skill* OR knowledge OR competen* OR scope-of-practice OR education* OR training*)).ti,ab,kf. | 200,076 |
| #1 | Palliative Care/ or Palliative Medicine/ or Terminal Care/ or "Hospice and Palliative Care Nursing"/ or (palliative or terminal or hospice or limited-life or end-of-life).ti,ab,kf. | 563,472 |

### Cinahl (Ebsco) History and Search Details – 20 July 2022

| **Search** | **Query** | **Results** |
| --- | --- | --- |
| S7 | Refine by: Academic Journals | 3,681 |
| S6 | S4 NOT TI (undergraduate OR medical-student* OR nurs*-student* OR family-care* OR informal-care* OR covid OR oncol*) | 4,058 |
| S5 | S4 – LIMITERS Published Date: 20100101- | 4,702 |
| S4 | S3 NOT ((MH ("Adolescence" OR "Child+") OR TI (adolescen* OR child* OR schoolchild* OR infant* OR girl* OR boy* OR teen OR teens OR teenager* OR youth* OR pediatr* OR paediatr* OR puber*) OR AB (adolescen* OR child* OR schoolchild* OR infant* OR girl* OR boy* OR teen OR teens OR teenager* OR youth* OR pediatr* OR paediatr* OR puber*)) NOT (MH ("Adult+") OR TI (adult* OR man OR men OR woman OR women) OR AB (adult* OR man OR men OR woman OR women))) | 7,058 |
| S3 | S1 AND S2 | 7,414 |
| S2 | (MH ("Specialization" OR "Nurse Practitioners+" OR "Caregivers" OR "Physicians+") AND MH ("Role" OR "Professional Role+" OR "Professional Competence+" OR "Education+" OR "Education, Nursing+" OR "Education, Medical+")) OR TI ((specialist* OR expert* OR non-expert* OR nurs* OR caregiver* OR care-giver* OR physician*) N3 (role OR roles OR defin* OR distin* OR characteri* OR skill* OR knowledge OR competen* OR scope-of-practice OR education* OR training*)) OR AB ((specialist* OR expert* OR non-expert* OR nurs* OR caregiver* OR care-giver* OR physician*) N3 (role OR roles OR defin* OR distin* OR characteri* OR skill* OR knowledge OR competen* OR scope-of-practice OR education* OR training*)) | 187,779 |
| S1 | MH ("Palliative Medicine" OR "Palliative Care" OR "Hospice and Palliative Nursing" OR "Terminal Care+") OR TI (palliative OR terminal OR hospice OR limited-life OR end-of-life) OR AB (palliative OR terminal OR hospice OR limited-life OR end-of-life) | 111,680 |

### Web of Science Core Collection History and Search Details – 20 July 2022

| **Search** | **Query** | **Results** |
| --- | --- | --- |
| #6 | #5 NOT TI= (undergraduate OR medical-student* OR nurse-student* OR family-care* OR informal-care* OR covid OR oncol*) | 3,333 |
| #5 | #4 Timespan: 2010-01-01 to 2023-12-31 (Publication Date) | 3,838 |
| #4 | #3 NOT (TI=(adolescent* OR child* OR schoolchild* OR infant* OR girl* OR boy* OR teen OR teenager* OR youth* OR pediatr* OR paediatr* OR puber*) NOT TI=(adult* OR “man” OR “men” OR woman OR women)) | 4,925 |
| #3 | #1 AND #2 | 5,166 |
| #2 | TS = ((specialist* OR expert* OR non-expert* OR nurs* OR caregiver* OR care-giver* OR physician*) NEAR/3 (role OR roles OR defin* OR distin* OR characteri* OR skill* OR knowledge OR competen* OR scope-of-practice OR education* OR training*)) | 152,443 |
| #1 | TS = (palliative OR terminal OR hospice OR limited-life OR end-of-life) | 690,557 |
