# Supplementary material for: Specialized expertise among healthcare professionals in palliative care - A scoping review
Source: BMC Palliat Care. 2024 Jul 13;23:170. doi: 10.1186/s12904-024-01498-0 (PMC11245811; doi:10.1186/s12904-024-01498-0)
Supplement: Supplementary file 2 — Supplementary Material 2 [file 12904_2024_1498_MOESM2_ESM.docx]

**Additional file 2: Table S1. Included study characteristics.**

| **Table S1. Included study characteristics** | | | | | |
| --- | --- | --- | --- | --- | --- |
| **1st author** | **Year** | **Objective** | **Study design** | **Population** | **Country** |
| Abbasi | 2019 | To improve palliative care organization through (interdisciplinary) guidelines. | Edited interview on palliative care guidelines | Physicians | United States |
| Atreya | 2019 | To outline the palliative care competencies required for a primary care/family physician. | Competence framework paper | Physicians | India |
| Autelitano | 2021 | To present a specialist profile of palliative care nurses. | Organizational case study approach | Nurses | Italy |
| Ballou | 2021 | To include formal palliative care education on structured communication and prognostication in Surgical Education | Peer-reviewed narrative article | Physicians | United States |
| Boddaert | 2021 | To identify characteristics of hospital palliative care programs and their Specialist Palliative Care Teams. | Secondary analysis of a national cross-sectional survey | Palliative care program leaders of hospitals | The Netherlands |
| Brown | 2018 | To describe the models of physician-based palliative care services delivered to patients in the last 12 months of life. | Retrospective cohort study | Physicians  Patient cohort (*n* = 361,951) | Canada |
| Carroll | 2018 | To measure the perceived importance of primary palliative care topics and preferences about learning settings. | Cross sectional study using a survey | Physicians (*n* = 260) | The United States of America |
| Centeno | 2015 | To provide a detailed, comparative update and analysis of the palliative medicine certification process in Europe. | Comparative analysis of data from an online survey | Palliative medicine experts (*n* = 16) | Italy |
| Chang | 2016 | To investigate the content needs of nurses with regard to a palliative care in-service education program. | Cross-sectional study using a survey | Nurses (*n* = 600) | Taiwan |
| Chen | 2022 | To evaluate the effects of a training program on nurses’ knowledge and attitudes to palliative care. | Cross-sectional survey | Nurses (*n* = 10,048) | China |
| Chovan | 2013 | To evaluate the structures and processes of an inpatient palliative care consultation service in light of national guidelines. | Cross-sectional case study | Palliative care professionals (*n* = 7) | The United States of America |
| Connolly | 2016 | To develop a Palliative Care Competence Framework for health and social care professionals working in the Republic of Ireland. | Report on development of A Palliative Care Competence Framework | Health and social care professionals | Ireland |
| Connolly | 2018 | To describe assess the level of competence of medical doctors in Ireland to provide palliative care. | Survey-based cohort study | Physicians/trainees (*n* = 328) | Ireland |
| Connolly | 2021 | To evaluate knowledge and perceptions of the role of the specialist palliative care clinical nurse specialist (SPC CNS) in an acute hospital setting. | Mixed methods sequential explanatory study | Nurses and physicians (*n* = 231) | Ireland |
| Deitrick | 2011 | To understand and create a model of the OACIS (Optimizing Advanced Complex Illness Support) Nurse Practitioner role. | Theoretical review and Grounded Theory approach | Nurse Practitioners | The United States of America |
| Engel | 2021 | To describe views of palliative care nurse champions. | Qualitative interview study | Nurses (*n* = 6) | The Netherlands |
| Firn | 2016 | To assess the existing evidence of inpatient generalist palliative care providers’ perceptions of what facilitates or hinders collaboration with hospital-based specialist palliative care. | Narrative literature synthesis with systematically constructed search | Nurses and Physicians | The United States of America |
| Fischer | 2021 | To evaluate the implementation of a new Nurse Practitioner role within an established multidisciplinary palliative care service. | Cross-sectional study using a survey | Nurse Practitioners  Patients (*n* = 1,015) | Australia |
| Forbat | 2020 | To achieve consensus regarding what distinguishes specialist from non-specialist palliative care to inform service organization and delivery to patients with life-limiting conditions. | Three-phase Delphi study | Nurses, physicians and social workers (*n* = 31) | Australia |
| Frey | 2011 | To ascertain what questionnaire tools exist to measure the perceived competence of generalists in palliative care provision. | Systematic review | Palliative care clinicians | New Zealand |
| Gardiner | 2012 | To explore factors that support partnership working between specialist and generalist palliative care providers. | Systematic literature review | Palliative care clinicians | United Kingdom |
| Gardiner | 2022 | To explore perspectives CNSs on their role in providing palliative care for patients with mesothelioma. | Qualitative study | Clinical Nurse Specialists (*n* = 31) | United Kingdom |
| Hokka | 2020 | To give evidence of nursing competencies needed in palliative care and whether these competencies differ per level. | Systematic integrative literature review with thematic synthesis | Nurses | Finland |
| Hokka | 2021 | To describe the required palliative nursing competencies of registered nurses aligned to different levels of palliative care provision. | Qualitative study | Nurses (*n* = 222) | Finland |
| Howell | 2014 | To describe community palliative care clinical nurse specialist activities during interactions with patients. | Qualitative study | Nurses (*n* = 4) | United Kingdom |
| Imhof | 2016 | To describe the characteristics of a specialist nurse-led palliative care service in an urban Swiss region. | Qualitative study | Interdisciplinary palliative home care professionals (*n* = 41) | Switzerland |
| Kang | 2013 | To report the results of developing the hospice and palliative care competencies. | Delphi study | Palliative care professionals (*n* = 42) | Korea |
| Klarare | 2013 | To explore team interaction in specialized palliative care teams. | Qualitative study | Palliative care professionals (*n* = 15) | Sweden |
| Maher | 2013 | To explore when palliative care specialists are needed. | Qualitative study using a panel discussion | Nurses | Australia |
| Melender | 2022 | To describe the most essential competencies of palliative-care nurses and physicians. | A prospective cross-sectional study, survey | Nurses (*n* = 129) and physicians (*n* = 64) | Finland |
| Mulvihill | 2010 | To obtain an improved understanding of the role defined in the literature as the specialist palliative care community nurse. | Literature review | Nurses | Australia |
| Quill | 2013 | To create a more sustainable model for generalists and specialists in palliative care. | Discussion paper | Nurses and physicians | The United States of America |
| Robinson | 2017 | To explore the influence of philosophy on nurses palliative care model. | Discussion paper | Nurses | The United Kingdom |
| Runacres | 2021 | To examine the knowledge, attitudes and practices of geriatricians in providing and working with specialist palliative care. | A prospective cross-sectional study, survey | Physicians (*n* = 168) | Australia and New Zealand |
| Ryan | 2014 | To articulate our understanding of discipline-specific and inter-professional competences within palliative care. | Framework report | Palliative care clinicians | Ireland |
| Salins | 2016 | To review integration of early specialist palliative care in cancer care. | Literature review | Patients, palliative care professionals | India |
| Sakashita | 2019 | To develop a Japanese national consensus syllabus of palliative medicine for physicians. | Modified Delphi study | Physicians | Japan |
| Wikert | 2022 | To develop an comprehensive classification to facilitate the description and differentiation of specialist palliative care models. | Qualitative study | Nurses and physicians (*n* = 27) | Germany |
